# Plutonium aided reconstruction of caesium atmospheric fallout in European topsoils

**DOI:** 10.1038/s41598-020-68736-2

**Published:** 2020-07-16

**Authors:** Katrin Meusburger, Olivier Evrard, Christine Alewell, Pasquale Borrelli, Giorgia Cinelli, Michael Ketterer, Lionel Mabit, Panos Panagos, Kristof van Oost, Cristiano Ballabio

**Affiliations:** 1 Swiss Federal Institute for Forest, Snow and Landscape Research (WSL), 8903 Birmensdorf, Switzerland; 2 Laboratoire des Sciences du Climat et de l’Environnement (LSCE-IPSL), UMR 8212 (CEA-CNRS-UVSQ), Université Paris-Saclay, CEA Saclay, l’Orme des Merisiers, 91191 Gif-sur-Yvette Cedex, France; 3 Environmental Geosciences, University of Basel, Bernoullistrasse 30, 4056 Basel, Switzerland; 4 European Commission, Joint Research Centre, Via E. Fermi 2749, 21027 Ispra, VA Italy; 5 Chemistry Department, Metropolitan State University of Denver, Denver, CO USA; 6 Soil and Water Management and Crop Nutrition Laboratory (SWMCNL), Joint FAO, IAEA Division of Nuclear Techniques in Food and Agriculture, Seibersdorf, Austria; 7 TECLIM, George Lemaitre Center for Earth and Climate, Earth and Life Institute, Université Catholique de Louvain, Louvain-La-Neuve, Belgium; 8 Fonds de la Recherche Scientifique (FNRS), 1000 Brussels, Belgium

**Keywords:** Environmental sciences, Solid Earth sciences, Physics

## Abstract

Global nuclear weapon testing and the Chernobyl accident have released large amounts of radionuclides into the environment. However, to date, the spatial patterns of these fallout sources remain poorly constrained. Fallout radionuclides (^137^Cs, ^239^Pu, ^240^Pu) were measured in soil samples (n = 160) collected at flat, undisturbed grasslands in Western Europe in the framework of a harmonised European soil survey. We show that both fallout sources left a specific radionuclide imprint in European soils. Accordingly, we used plutonium to quantify contributions of global versus Chernobyl fallout to ^137^Cs found in European soils. Spatial prediction models allowed for a first assessment of the global versus Chernobyl fallout pattern across national boundaries. Understanding the magnitude of these fallout sources is crucial not only to establish a baseline in case of future radionuclide fallout but also to define a baseline for geomorphological reconstructions of soil redistribution due to soil erosion processes.

## Introduction

Artificial fallout radionuclides (FRN) have been released into the environment during the atmospheric nuclear weapon tests that took place from the mid-1950s to 1980. After their emission in the stratosphere, they were mainly brought to the soils as a result of dry and wet fallout (i.e., rainfall and snowfall). The main long-lasting radionuclides of radioecological concern emitted during these tests, i.e. caesium-137 (^137^Cs; T_1/2_ = 30 years) and most plutonium isotopes including plutonium-239 (^239^Pu; T_1/2_ = 24,100 years) and plutonium-240 (^240^Pu; T_1/2_ = 6,560 years) are strongly particle-bound, and thus remain concentrated in the uppermost surface layer of the soil. The spatial pattern of global fallout is expected to follow latitudinal bands, in areas characterised by similar levels of precipitation^1^.

The overall distribution of radionuclides in European soils was substantially impacted after the most severe nuclear power plant (NPP) accident that took place in Chernobyl on April 26, 1986^2,3^. As radionuclides were emitted in lower atmospheric layers (i.e. troposphere), the Chernobyl radiocaesium deposition was much more heterogeneous across space than the global fallout because it originated from few distinct precipitation events that occurred late in April and early in May 1986 when the radioactive cloud travelled across the European continent. In contrast, the second major NPP accident in history, the Fukushima Dai-ichi accident that occurred in 2011, only caused little fallout in Europe^4,5^.

Almost 80 years after the initial global deposition of artificial FRN, the magnitude and the spatial pattern of the Chernobyl versus the global radioactive fallout remains of broad societal and scientific interest but is highly uncertain^6^, and an assessment of their inventory at the continental scale is needed.

Following the Chernobyl accident, several research groups and monitoring facilities across Europe measured radioactivity levels. The reported levels of deposition were based on airborne gamma surveys, in situ measurements of gamma dose rates and spectrometry, and measurements of soil profiles (often collected at different depths). This heterogeneous dataset provided by national contact points formed the basis for drawing the “Atlas of cesium deposition on Europe after the Chernobyl accident”^6,7^. Only recently an updated database on deposition measurement over Europe was made available^8^. For example, on the national scale, a detailed map of Austria soil caesium inventory was produced^9^.

The existing post-Chernobyl radiocaesium deposition maps show the ^137^Cs irrespective of the fallout source^6,8^. Radionuclide inventories (also termed inventory or areal activity) in soils are conventionally expressed in Bq m^−2^ and decay-corrected to a given reference date for comparison. The assessment of the ^137^Cs deposition that occurred prior to the Chernobyl NPP accident is associated with even higher uncertainties, as no detailed assessments of the global fallout were conducted prior to the Chernobyl NPP. The only information currently available on the global ^137^Cs deposition levels was derived from strontium-90 (^90^Sr) deposition maps prepared by the United Nations Scientific Committee on the Effects of Atomic Radiation (UNSCEAR)^1,10^. However, ^90^Sr has the disadvantage of being relatively mobile in the plant-soil system, entering considerable uncertainty in the estimations. Furthermore, only around 200 measurements for ^90^Sr were available worldwide to delineate global spatial patterns of radiocaesium fallout before the Chernobyl accident^10^.

Besides ^90^Sr, other FRN ratios are useful to identify the source of the fallout. For instance, at the time of the Chernobyl fallout, ^134^Cs was used to discriminate the two fallout sources in soils; however, due to its short half-life (2.01 years) ^134^Cs is not detectable anymore^8^. Today most promising for the reconstruction of fallout sources are the plutonium isotopes (^239^Pu, ^240^Pu, often expressed as ^239+240^Pu) that were released worldwide following the above-mentioned thermonuclear tests. In contrast, following the Chernobyl accident, these isotopes deposited only regionally in the surroundings of accidented NPPs in Eastern and Northern Europe, as they were contained in the non-volatile fraction of the nuclear fuel debris^11^. As a consequence, the geographic distribution of Chernobyl ^239+240^Pu fallout is restricted to those regions located closer to the power plant such as Russia, Ukraine, Belarus, Poland, the Baltic countries, and Scandinavia, whereas the major part of Europe did not receive measurable ^239+240^Pu Chernobyl fallout levels^12^. As such, and because global fallout has a distinct ^239+240^Pu to ^137^Cs activity ratio, it is possible to reconstruct the sources of radiocaesium.

To avoid the limitations associated with the previous studies which were mainly based on national surveys, the current research analysed ^137^Cs, ^239^Pu and ^240^Pu following a harmonised methodology across borders of European countries. We took advantage of an existing pan-European topsoil survey available across the entire European Union. In 2015, the spatial extent of this survey was extended to include Switzerland and the Balkan countries, where topsoil samples were collected following a harmonised protocol (the so-called Land Use/Cover Area frame Survey—LUCAS—initiated by the European Commission Joint Research Centre)^13^. This study focuses on a selection of soil samples collected in France, Belgium, Southern Germany, Switzerland and Northern Italy, to cover regions, which are assumed to have received variable levels of Chernobyl radionuclide fallout. Radionuclide activities were measured with gamma- and mass spectrometry for topsoil profiles (0–20 cm) collected at reference sites (typically undisturbed, e.g. not ploughed, flat and permanent grassland), which were assumed not to be affected by soil redistribution processes. Then, based on relationships observed between those inventories and spatial covariates, we predict and analyse the patterns of global fallout, Chernobyl and total ^137^Cs and ^239+240^Pu fallout.

## Results

### ^239+240^Pu sources in European topsoil samples: ^240^Pu/^239^Pu atom ratios

Since the start of the atmospheric nuclear weapon tests in the twentieth century, anthropogenic plutonium has become a ubiquitous element in the environment, although it is generally found naturally at trace levels in uranium ores. Plutonium is of interest due to its high toxicity in addition to the potential associated radioactive threat but also represents a powerful tracer to partition radionuclide fallout sources. The atomic ratio ^240^Pu/^239^Pu provides discrimination between global fallout (^240^Pu/^239^Pu ca. 0.18)^16^ and Chernobyl nuclear reactor fallout (^240^Pu/^239^Pu ca. 0.4–0.6)^14^.

**Figure 1:**
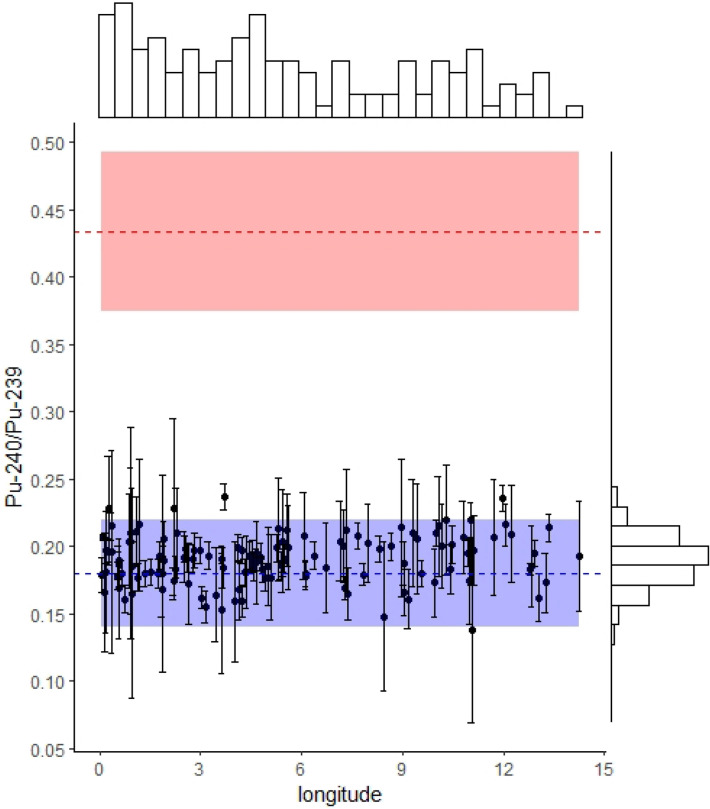
Atomic ratios of ^240^Pu to ^239^Pu measured in European topsoil samples (0–20 cm, n = 134). Error bars correspond to the standard deviation, which was calculated after conducting three sequential analyses of the same Pu extract solution from a single preparation of the sample. The analysed samples are normally distributed around the mean literature-derived global fallout ratio (dashed blue line = literature mean; blue ribbon = literature derived standard variation). For comparison, the literature-derived mean Chernobyl fallout atom ratio is displayed (dashed red line = literature mean; red ribbon = literature derived standard variation). Histograms at the top and right side represent the distribution of the samples.

For the topsoil samples (n = 134) analysed in the current research, the ratios of ^240^Pu/^239^Pu are normally distributed around a mean value of 0.19 ± 0.018 (standard deviation; SD). Within the range of the measurement uncertainty, the atomic ratio of all samples corresponds to the global fallout signature (Fig. 1). In case of presence of Chernobyl-derived ^239+240^Pu, we would expect a positive correlation to Chernobyl-derived ^137^Cs, which was not the case (Supplementary Fig. 1). The ^239+240^Pu found in soils of the studied western part of Europe, therefore, originated predominantly from global fallout, which allows using the ^239+240^Pu/^137^Cs activity ratio for discrimination between the two potential sources of ^137^Cs^11^.

### ^137^Cs sources in European topsoil samples: ^240+239^Pu/^137^Cs activity ratios

Mean radionuclide mass activities measured in the topsoil samples amounted to 20.6 Bq kg^−1^ for ^137^Cs (SD: 30.4 Bq kg^−1^) and to 0.32 Bq kg^−1^ for ^239+240^Pu (SD: 0.27 Bq kg^−1^), those activities being decay-corrected to the soil sampling date (i.e. August 1st, 2009). Caesium and, to a lower extent, plutonium activities are right-skewed exhibiting some soils with relatively higher activities of up to 221 Bq kg^−1^ for ^137^Cs and 1.87 Bq kg^−1^ for ^239+240^Pu (Supplementary Fig. 2). The median activity corresponds to 11.4 Bq kg^−1^ with a mean absolute deviation (MAD) of 9.5 Bq kg^−1^ for ^137^Cs and 0.25 ± 0.15 (MAD) Bq kg^−1^ for ^239+240^Pu. This high variability reflects the spatial heterogeneity of fallout.

**Figure 2:**
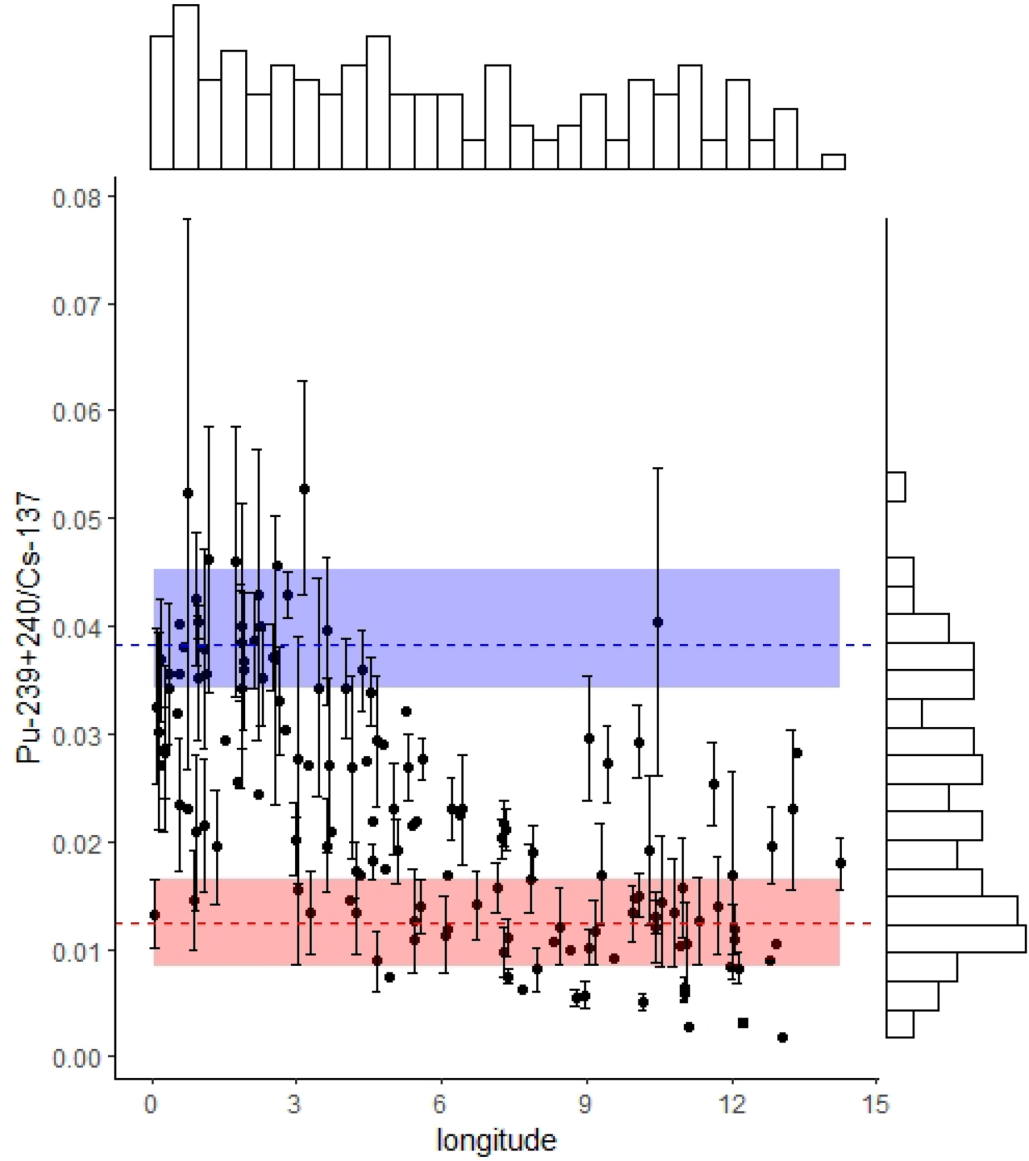
^239+240^Pu/^137^Cs activity ratio (black dots) for European topsoil samples (0–20 cm, n = 134) and literature-derived specific ratios for Chernobyl fallout (dashed red line = literature mean; red ribbon = literature derived standard variation) and the global fallout (dashed blue line = literature mean; blue ribbon = literature derived standard variation). Error bars correspond to the propagated error of the gamma spectrometry and ICP-MS measurements. Histograms at the top and right side represent the distribution of the samples.

The ^240+239^Pu/^137^Cs activity ratios are scattered between the specific ratios characterising the Chernobyl-(0.0124 ± 0.0016) and the global fallout (0.038 ± 0.004; both decay-corrected to August 1, 2009), reflecting the mixture of both sources in the soils (Fig. 2). The distribution of the activity ratios is slightly bimodal with peaks close to the specific ratios characterising the influence of both sources. When plotted against longitude, the activity ratios reflect a decrease eastwards indicating a higher contribution of Chernobyl derived ^137^Cs in the closer vicinity to the Chernobyl NPP accident (Fig. 2). Only a couple of the sites located between 10 and 15 degrees of longitude (Fig. 2) show lower Chernobyl contribution. These sites have likely been orographically protected from Chernobyl fallout such as in the inner Alpine dry valleys (n = 4). In contrast, a couple of sites at lower longitudes (located in western France) show a clear Chernobyl fallout signature. Not all activity ratio values lie within the expected range (Fig. 2). However, when taking into account the uncertainties the sites associated with ratios > 0.038 that received more ^239+240^Pu compared to ^137^Cs cannot be considered as outliers.

### Spatial patterns of ^239+240^Pu and ^137^Cs baseline fallout

**Figure 3:**
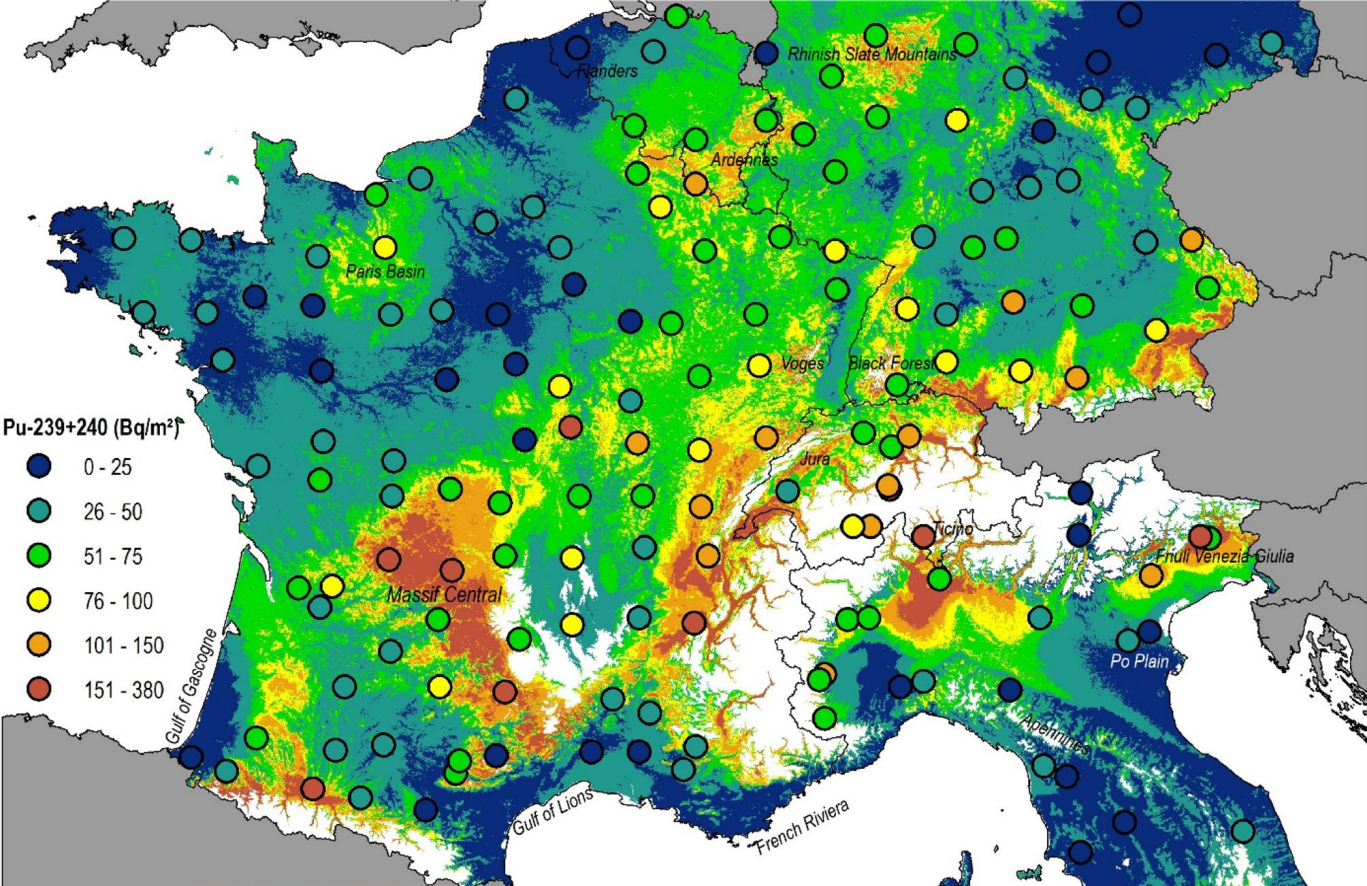
Baseline Plutonium-239 + 240 topsoil (0–20 cm) inventories (Bq m^−2^) estimated with a generalised additive model with a resolution of 500 m. Dots represent those Plutonium-239 + 240 inventories measured at potentially undisturbed reference sites (flat grassland soils). The same colour range applies for the dots and the interpolated map. In the study area, areas above 1,000 m a.s.l. have been masked (white) using the publicly available SRTM digital elevation model at 3 arcsec (https://www2.jpl.nasa.gov/srtm/). The map (projection: ETRS89-Lambert Azimuthal Equal Area) is own compilation, using ESRI ArcGIS 10.5 Desktop. The national boundaries are Intellectual Property from European National Mapping and Cadastral Authorities and is licensed on behalf of these by EuroGeographics. Original product is available for free at www.eurogeographics.org. Terms of the licence available https://eurogeographics.org/maps-for-europe/open-data/topographic-data/.

Measured ^239+240^Pu activities converted into topsoil (0–20 cm) inventories range from 8.0 to 380.2 Bq m^−2^, and their median value amounted to 53.3 Bq m^-2^ (MAD: 31.5 Bq m^−2^, Supplementary Fig. 2). The spatial variability of the inventories could best (R^2^adj. = 0.70) be explained with the spatial XY-coordinates and the following covariates: mean rainfall during period 1952–1980 (which is representative of the global fallout period), mean annual average precipitation in December, the minimum and maximum temperatures in November and valley height due to their connection to orographic processes (Supplementary Table 1). The resulting residues are normally distributed (Supplementary Fig. 2). The predicted ^239+240^Pu inventories range from 4.9 to 616.3 Bq m^−2^ with a median of 46.6 Bq m^−2^ and a mean of 57.0 ± 41.4 Bq m^−2^. The highest ^239+240^Pu values were found in areas with high average annual precipitation such as the Alps, the Massif Central and lower highland areas such as the Jura, the Ardennes and the Rhinish Slate Mountains (Fig. 3). Orographic uplift and upwind and downwind patterns are clearly visible. Lower values are found in Eastern Germany, the Paris Basin and, close to the coast in the Gulf of Lions and Gascogne, the French Riviera and Flanders. No unidirectional trend is visible for latitude nor longitude in the studied area.

**Figure 4:**
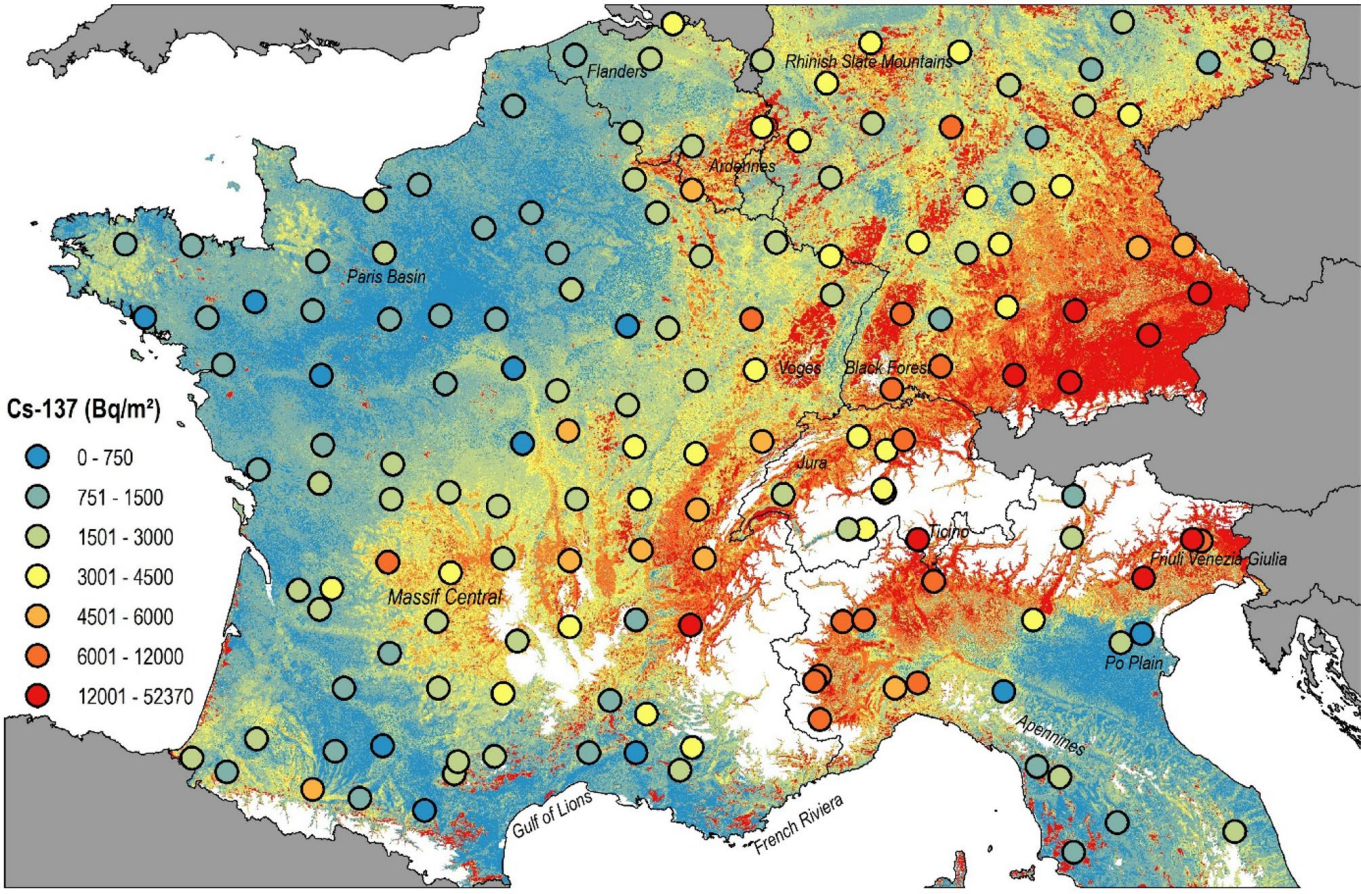
Baseline Caesium-137 topsoil (0–20 cm) inventories (Bq m^−2^) decay-corrected to August 1, 2009, estimated with a generalised additive model with a resolution of 500 m. Dots represent those Caesium-137 inventories measured at reference sites (flat, grassland soils). The same colour range applies for the dots and the interpolation. In the study area, areas above 1,000 m a.s.l. have been masked (white) using the publicly available SRTM digital elevation model at 3 arcsec (https://www2.jpl.nasa.gov/srtm/). The map (projection: ETRS89-Lambert Azimuthal Equal Area) is own compilation, using ESRI ArcGIS 10.5 Desktop. The national boundaries are Intellectual Property from European National Mapping and Cadastral Authorities and is licensed on behalf of these by EuroGeographics. Original product is available for free at www.eurogeographics.org. Terms of the licence available https://eurogeographics.org/maps-for-europe/open-data/topographic-data/.

The ^137^Cs inventories, when recalculated to 2009, amounted to median values of 2,349 Bq m^−2^ with a MAD of 1,975 Bq m^−2^. The distribution of the ^137^Cs inventories is right-skewed as for the activities, with the highest values of 52,370 Bq m^−2^ and 35,920 Bq m^−2^ measured in Ticino (Southern Switzerland) and Friuli Venezia Giulia (Northern Italy), respectively (Fig. 4). The lowest inventories < 600 Bq m^−2^ were measured in the lower part of the Po plain in Northern Italy (Fig. 4). The spatial distribution model could well predict the measured ^137^Cs inventories, including these extremely high inventory values (R^2^ = 0.96). The covariates selected by the model were mean rainfall during the period 1952–1980, rainfall in May 1986, the valley height, and as a proxy for vegetation cover the 4th PCA component of the near-infrared band and the 1st PCA component of the red band of Moderate Resolution Imaging Spectroradiometer (MODIS) data and the XY-coordinates. As observed for ^239+240^Pu, hotspots with high fallout levels are observed in pre-alpine and highland areas (e.g. Ardennes, Vosges, Black Forest). Overall, there is a clear increasing trend of ^137^Cs inventories towards eastern directions with the lowest values found in north-western France and the highest values predicted in south-eastern Germany and the southern side of the Alps.

### Global versus Chernobyl distribution of ^137^Cs inventories

**Figure 5:**
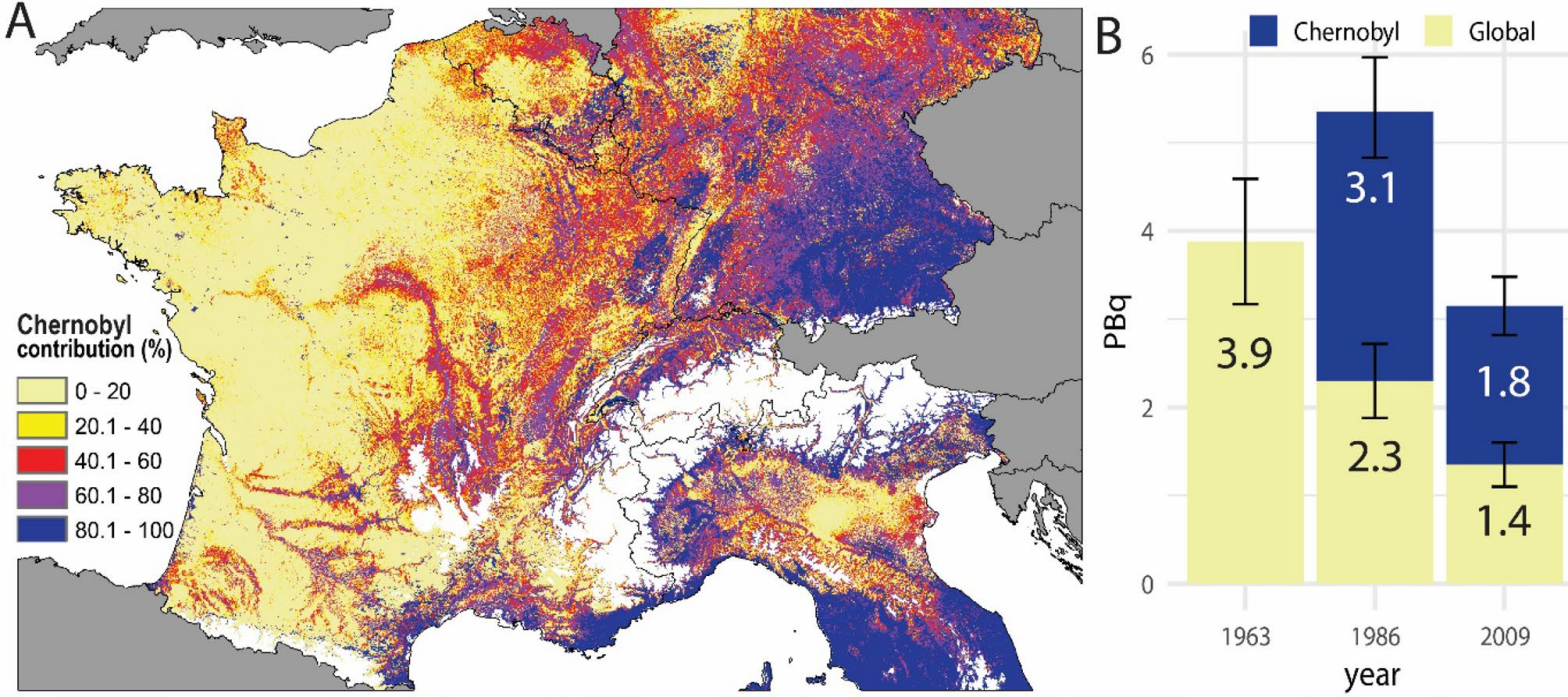
Chernobyl-derived ^137^Cs fallout contribution (%) of topsoil (0–20 cm) samples decay-corrected to August 1, 2009. The contribution map is derived from the regionalised total and global ^137^Cs fallout map (**A**). Source contribution of ^137^Cs (PBq) in western European topsoils (**B**). In the study area, areas above 1,000 m a.s.l. have been masked (white) using the publicly available SRTM digital elevation model at 3 arcsec (https://www2.jpl.nasa.gov/srtm/). The map (projection: ETRS89-Lambert Azimuthal Equal Area) is own compilation, using ESRI ArcGIS 10.5 Desktop. The national boundaries are Intellectual Property from European National Mapping and Cadastral Authorities and is licensed on behalf of these by EuroGeographics. Original product is available for free at . Terms of the licence available.

The Chernobyl derived ^137^Cs fallout contribution varies greatly within the studied western part of Europe with values higher than 80% (decay-corrected to August 1, 2009) for southeast Germany, the mid-mountain ranges and along the Alpine Arch (Fig. 5). In contrast, in large parts of France, the contributions are lower than 20%, and the ^137^Cs fallout is still dominated by the global fallout pattern (Fig. 5A). According to the spatial models, in the studied western part of Europe (1,320,407 km^2^), the total global-derived ^137^Cs corresponds to 3.9 ± 0.7 PBq (10^15^ Bq) in 1963 (taken as the reference date for the fallout period). The global-derived ^137^Cs decayed to 2.3 ± 0.4 PBq in 1986 when an additional 3.1 ± 0.6 PBq was supplied by the Chernobyl accident (Fig. 5B). By 2009, the contribution of the global fallout (1.4 ± 0.3 PBq) was lower than that of the Chernobyl accident (1.8 ± 0.3 PBq) although it supplied 44% ± 18.3% of the total ^137^Cs inventory found in soils of the western part of Europe (Fig. 5B). The suggested approach to calculate the Chernobyl ^137^Cs involves uncertainties that were propagated through Eqs. 1 and 2 and are displayed as upper and lower boundary maps (Supplementary Fig. 3).

The ^239+240^Pu/^137^Cs activity ratio derived as mean from those values found in the literature (Table 1) amounts to 0.038 ± 0.004 (^137^Cs/^239+240^Pu = 26.2, decay corrected to the 1^st^ of August 2009). Multiplying the activity ratio with the ^239+240^Pu activities provides a way to calculate the proportion of the ^137^Cs fallout activities originating from the global fallout, which were converted into inventories and subsequently regionalised.

**Table 1:** Analytical results (bold) and a literature survey of ^240^Pu/^239^Pu atom ratios and ^240+239^Pu/^137^Cs activity ratios in soils (decay-corrected to 1.8.2009).

Atom ratio of ^240^Pu/^239^Pu						
Reference	Sample	^240^Pu/^239^Pu	SD	Pu Inv	Location	Source
IAEA^17^	–	0.380	–	–	–	Chernobyl
Belyayev et al.^18^	n = 3 soil samples	0.350	–	–	10-km zone Chernobyl	Chernobyl
Belyayev et al.^18^	n = 3 soil samples	0.303	–	–	30-km zone Chernobyl	Chernobyl
Begichev et al.^19^	Estimated from reactor core	0.425	–	–	Chernobyl reactor	Chernobyl
Boulyga et al.^20^	Single hot particle	0.330	0.05	–	Chernobyl reactor	Chernobyl
Krüger et al.^21^	–	0.430	–	–	–	Chernobyl
Erdmann et al.^22^	Several soil samples	0.377	0.037	–	Chernobyl	Chernobyl
Kirchner and Noack^23^	Estimated from reactor core	0.560	–	–	–	Chernobyl
Muramatsu et al.^14^	n = 3 soil samples, topsoil	0.408	0.003	6,647	10-km zone Chernobyl	Chernobyl
Muramatsu et al.^14^	n = 5 soil samples, topsoil	0.401	0.006	634	30-km zone Chernobyl	Chernobyl
Ketterer et al.^11^	n = 2 peat and n = 5 forest soils	0.186 to 0.348	–	–	Poland	Chernobyl, Global
Everett et al.^24^	n = 20 soils samples, depth 1 cm	0.149	0.003	–	Australia	Global
Kelley et al.^25^	n = 24 soil samples, depth 0–30 cm	0.180	0.014	–	71–30 N	Global
Kelley et al.^25^	n = 7 soil samples, depth 0–30 cm	0.178	0.019	–	0–30 N	Global
Kelley et al.^25^	n = 8 soil samples, depth 0–30 cm	0.173	0.027	–	0–30 S	Global
Kelley et al.^25^	n = 9 soil samples, depth 0–30 cm	0.185	0.047	–	30–53 S	Global
Meusburger et al.^26^	n = 7 soil profiles	0.182	0.015	55	South Korea	Global
Meusburger et al.^27^	n = 10 soil profiles	0.183	0.013	67	Swiss Alps	Global
Tims et al.^28^	n = 3 soil profiles	0.130	0.004	8.8	Australia	Global
Xu et al. ^29^	n = 6, surface soils	0.192	0.033	86.9–89	Northeast China	Global
**This study**	**n = 160 soil samples, depth 0–20 cm**	**0.190**	**0.021**	**53**	**Europe**	**Global**

Activity ratio of ^239+240^Pu/^137^Cs						
Reference	Sample	^239+240^Pu/^137^Cs*	SD	Cs Inv	Location	Source
Muramatsu et al.^14^	n = 3 soil samples	0.008	0.0003	799,000	10-km zone Chernobyl	Chernobyl
Muramatsu et al.^14^	n = 5 soil samples	0.010	0.0009	61,140	30-km zone Chernobyl	Chernobyl
Meusburger et al.^27^	n = 10 soil profiles	0.010	0.005	7,421	Central Swiss Alps	mainly Chernobyl
Mitchell et al.1990*	n = 11 soil samples, depth 0–30 cm	0.036	0.002	1,985	Ireland	Chernobyl, Global
Hardy^30^	n = 11 soil samples	0.038	0.001	–	US-wide	Global
Hodge et al.^31^	n = 15 soil samples, depth 6.4 cm	0.037	0.003	–	Denver, Colorado	Global
Kim et al.^32^**	n = 27 soil samples, depth 0–5 cm	0.033	0.003	–	South Korea	Global
Krey and Beck^33^	n = 13 soil samples	0.036	0.003	–	Utah	Global
McArthur and Miller^34^	n = 66 Soil samples	0.039	0.003	–	US-wide	Global
Meusburger et al.^26^	n = 7 soil profiles	0.029	0.007	1,607	South Korea	Global
Peirson et al.^35^	n = 64 soil samples	0.035	0.004	–	Scotland	Windscale, Global
**This study**	**n = 160 soil samples, depth 0–20 cm**	**0.023**	**0.011**	**2,349**	**Europe**	**Chernobyl, Global**

Pu and Cs Inv refers to the reported inventory (Bq m^−2^).

*For Cs-137 no decay correction date was provided. For comparison, it was decay-corrected to 1988, which corresponds to the year of sampling.

**For Cs-137 no decay correction date was provided. For comparison, it was decay-corrected to 1995, which corresponds to the year of sampling.

**Figure 6:**
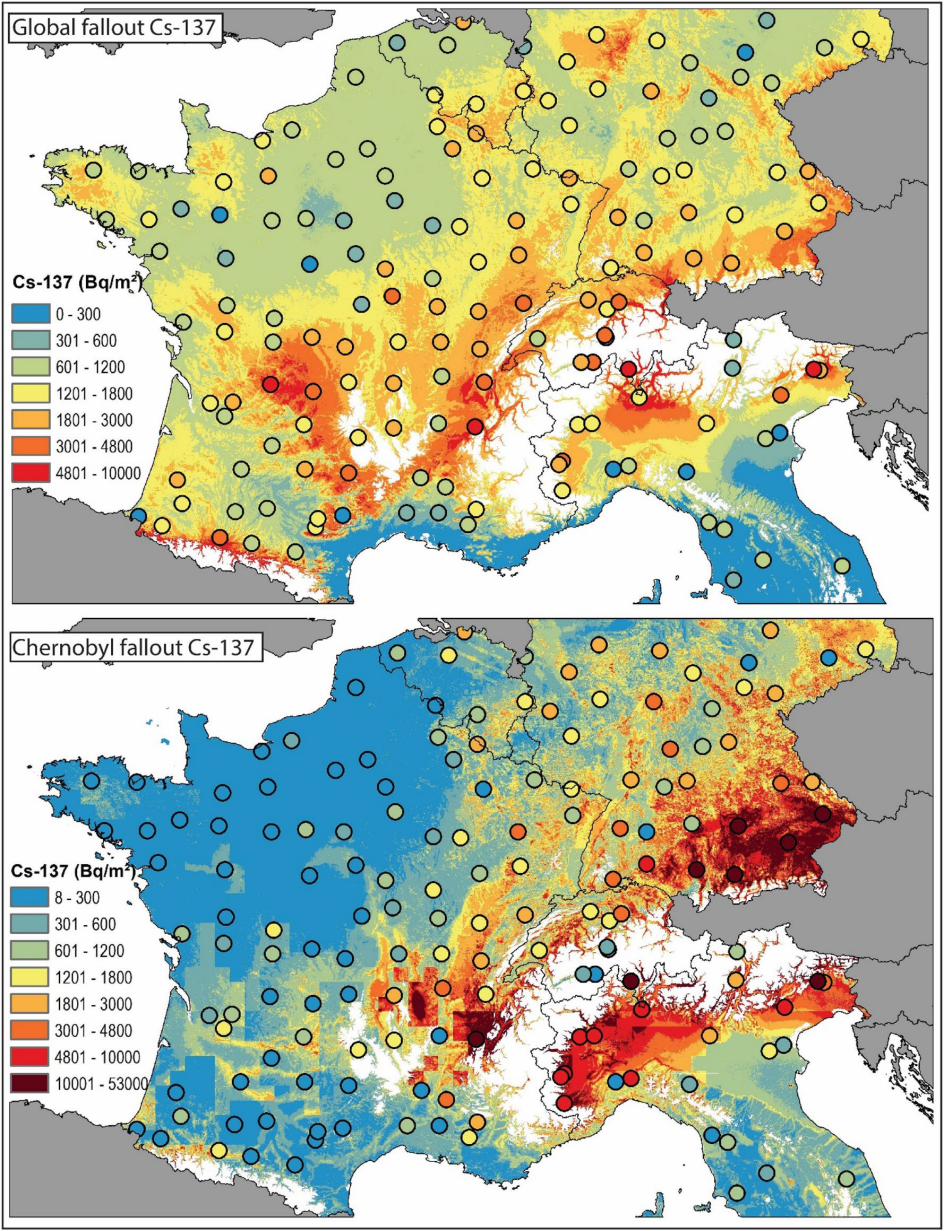
Global- and Chernobyl-derived ^137^Cs topsoil (0–20 cm) inventories (Bq m^−2^) decay- corrected to August 1, 2009, and interpolated with a general additive model at a resolution of 500 m. Dots represent those ^37^Cs inventories measured at reference sites (flat, grassland soils). The same colour range applies for the dots and the raster grids. In the study area, areas above 1,000 m a.s.l. have been masked (white) using the publicly available SRTM digital elevation model at 3 arcsec (https://www2.jpl.nasa.gov/srtm/). The map (projection: ETRS89-Lambert Azimuthal Equal Area) is own compilation, using ESRI ArcGIS 10.5 Desktop. The national boundaries are Intellectual Property from European National Mapping and Cadastral Authorities and is licensed on behalf of these by EuroGeographics. Original product is available for free at www.eurogeographics.org. Terms of the licence available https://eurogeographics.org/maps-for-europe/open-data/topographic-data/.

Following this calculation method, the resulting spatial pattern of the prior Chernobyl global-derived ^137^Cs fallout shows a substantial similarity with the ^239+240^Pu inventory map (Fig. 6). In the analysed topsoil samples, the global fallout-derived ^137^Cs inventories range from 210 Bq m^−2^ to 9,962 Bq m^−2^, with a median value of 1,395 Bq m^−2^. For the spatial interpolation of global-derived ^137^Cs activities, the same set of covariates as selected for Pu achieved the best prediction (R^2^ = 0.7). Similar as observed for the ^239+240^Pu values, the highest global ^137^Cs fallout occurred in areas exposed to high levels of precipitation (Fig. 6).

In contrast, the Chernobyl-derived ^137^Cs inventory, calculated as the difference between the total and the global ^137^Cs inventory, is characterised by a distinct east–west gradient reflecting the rainfall patterns that prevailed during the weeks that followed the Chernobyl accident and the radioactive cloud pathway across Europe (Fig. 6). In addition to the XY-coordinates, the valley height and the 4th component of the MODIS near-infrared band, the mean rainfall in May 1986 together with the daily rainfall amounts on May 2nd and 3rd, 1986, were selected as the main covariates to predict the observed Chernobyl-derived ^137^Cs inventory (R^2^ = 0.97). The occurrence of rainfall during these two days in May 1986 likely explains the higher Chernobyl-derived ^137^Cs inventories predicted in the Garonne River basin, in southwestern France. Overall, the Chernobyl fallout is characterised by significant gradients across space while the global fallout is, as expected, more evenly distributed. Both fallout source distribution maps are also characterised by contrasting direction patterns. For instance, in France, west exposed slopes of mountain ranges were more affected by the global fallout, whereas the Chernobyl fallout was higher on the eastern side of the mountain ranges (Fig. 6). Hotspots of Chernobyl ^137^Cs fallout were predicted near the Alps and particularly in southeastern Germany. In contrast, there was virtually no Chernobyl fallout in soils of northwestern France.

## Discussion

The ^240^Pu/^239^Pu ratios reflect the exclusive global fallout origin of the ^239+240^Pu found in soils of Western Europe since the analysed ratios remain in excellent agreement with those found in reference locations worldwide reported in the literature (Table 1). Based on this crucial prerequisite, the ^239+240^Pu/^137^Cs activity ratio is suitable to partition the respective contributions of the Chernobyl-versus the global fallout-derived ^137^Cs found in soils.

Some sites that received high ^137^Cs fallout levels, i.e. with inventories > 20,000 Bq m^−2^, showed ^239+240^Pu/^137^Cs activity ratios that remained well below those observed in the zone located within a radius of 10–30 km around the Chernobyl NPP (red ribbon, Fig. 2). Uncertainty of gamma spectrometry measurements provides the main contribution to the overall uncertainty associated with the activity ratio, and it becomes higher when ^137^Cs activities are low. This is evident by large error bars (Fig. 2) in those sites that received a limited amount of Chernobyl fallout. However, measurement uncertainty cannot explain the values lower than the ratio associated with the Chernobyl fallout that reflects a very high ^137^Cs activity compared to the global ^240+239^Pu fallout level (extremely narrow error bars, Fig. 2). High Chernobyl fallout sites located in zones of orographic uplift were well predicted with the rainfall covariates and, accordingly, these high inventories are unlikely to be outliers nor a result from soil profile disturbance or mixing, e.g. soil accumulation due to sedimentation or a local fallout source. An alternative explanation for these low ratio values might be a loss of ^239+240^Pu to deeper (> 20 cm) soil layers due to migration processes. Migration velocity of Pu in mineral soils largely depends on its oxidation state. Generally, humic acids can reduce Pu(VI) or Pu(V) to Pu(IV) and might provide an important path of plutonium immobilization^15,16^. However, this assumption of higher Pu migration with depth is unlikely as the sites with high inventories were characterised by high organic carbon contents and pH values of 5 to 7.5, and Cs/Pu migration remains limited in these conditions. In general, soil properties of sites below the range of Cs/Pu rates and within the range did not differ significantly (t-test). Furthermore, these soils with low ratios cumulated both the highest ^137^Cs and ^239+240^Pu inventories and both radionuclides are known to be strongly particle-bound, and their migration with depth is expected to remain therefore very limited. More detailed discussion on the uncertainties of the approach is provided in the “Materials and methods” section.

### Similarity and deviations to previous fallout maps

In Europe, the primary source of ^137^Cs distribution before this study was the *Atlas of cesium deposition on Europe after the Chernobyl accident*^6,7^. The Atlas was based on deposition measurements from airborne gamma surveys, in situ measurements of gamma dose rates, spectrometry and measurements of soil profiles immediately after the Chernobyl accident. Our approach estimates the FRN fraction that remained bound to soil particles at sites that were not affected by soil disturbance nor mobilisation. Accordingly, this approach predicted potential baseline inventories in the absence of those disturbances. Deviations from the deposition map might, therefore, occur in areas where the radionuclide adsorption capacity of the soil was low, or the infiltration of rainfall was altered by surface flow patterns. The latter hypothesis is supported by the fact that the covariate valley height was significant for each model.

The global fallout ^137^Cs pattern derived from the ^239+240^Pu/^137^Cs ratio topsoil measurements shows a strong dependence on the rainfall covariates, which mainly results in the highest inventories found in mountain areas and other regions exposed to high precipitation levels. The only other references of prior Chernobyl ^137^Cs spatial distribution are the UNSCEAR report^1^. According to this UNSCEAR report, the average levels of global deposition follow latitudinal bands with ^137^Cs average levels of 1,055, 1,407, 1,290 Bq m^−2^ for the 30–40°N, 40–50°N and 50–60°N, respectively (all values being decay-corrected to August 1, 2009)^1^. These levels agree well with the median of 1,395 Bq m^−2^ found in this study. However, our approach shows for the first time that there is a considerable spatial variation of the global fallout derived ^137^Cs inventories mainly dependent on the precipitation pattern.

So far, very limited information was available about the global fallout distribution in Europe since the deposition pattern was derived from only a few samples analysed for ^90^Sr in 1971^6,10^. Although Sr^2+^-ions are also bound to the surface of clay minerals and humic substances, they are easily displaced by other alkaline ions, like Ca^2+^ and Mg^2+^. Therefore, compared to ^137^Cs, the migration velocities of ^90^Sr are much higher, with rates estimated to 5–25 cm/year^36^. Given the higher mobility of ^90^Sr in soils, its suitability to provide a proxy to reconstruct the global fallout pattern was likely limited.

**Figure 7:**
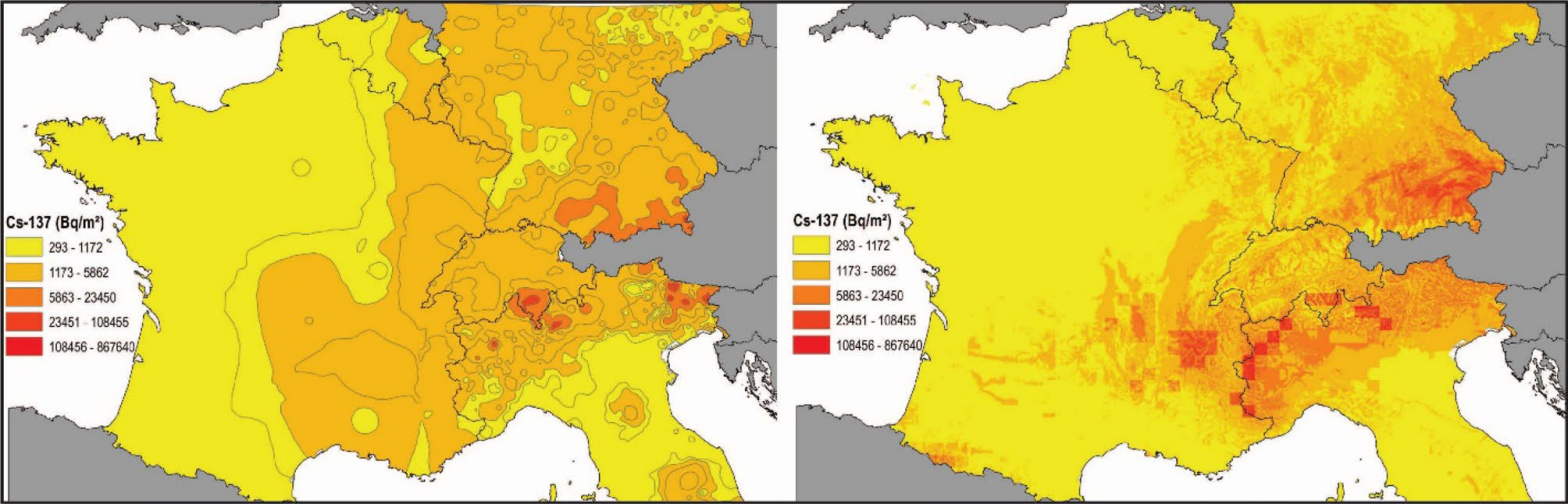
Comparison of post-Chernobyl ^137^Cs deposition map (Bq m^−2^) according to^6^. Copyright 1998 by EC/IGCE, Roshydromet(Russia)/Minchernobyl (Ukraine)/Belhydromet (Belarus). (left) and the topsoil inventories map produced in this study (right). All data are-decay corrected to August 1, 2009, and the same colour ranges were applied in both maps. The maps (projection: ETRS89-Lambert Azimuthal Equal Area) were produced using ESRI ArcGIS 10.5 Desktop. The national boundaries are Intellectual Property from European National Mapping and Cadastral Authorities and is licensed on behalf of these by EuroGeographics. Original product is available for free at www.eurogeographics.org. Terms of the licence available.

Comparison between the post-Chernobyl ^137^Cs spatial distribution provided by the Atlas (Fig. 7, left) and the predictions based on the LUCAS soil sample bank conducted in the current research (Fig. 7, right) results in higher levels of detail on those areas exposed to Chernobyl fallout in the new assessment. Furthermore, higher inventories were predicted for the mid-mountain regions as the Vosges, Black Forest and Ardennes as well as at the foot of the Pyrenees when comparing the updated map to that found in the Atlas.

### Comparison to local measured baseline inventories

**Figure 8:**
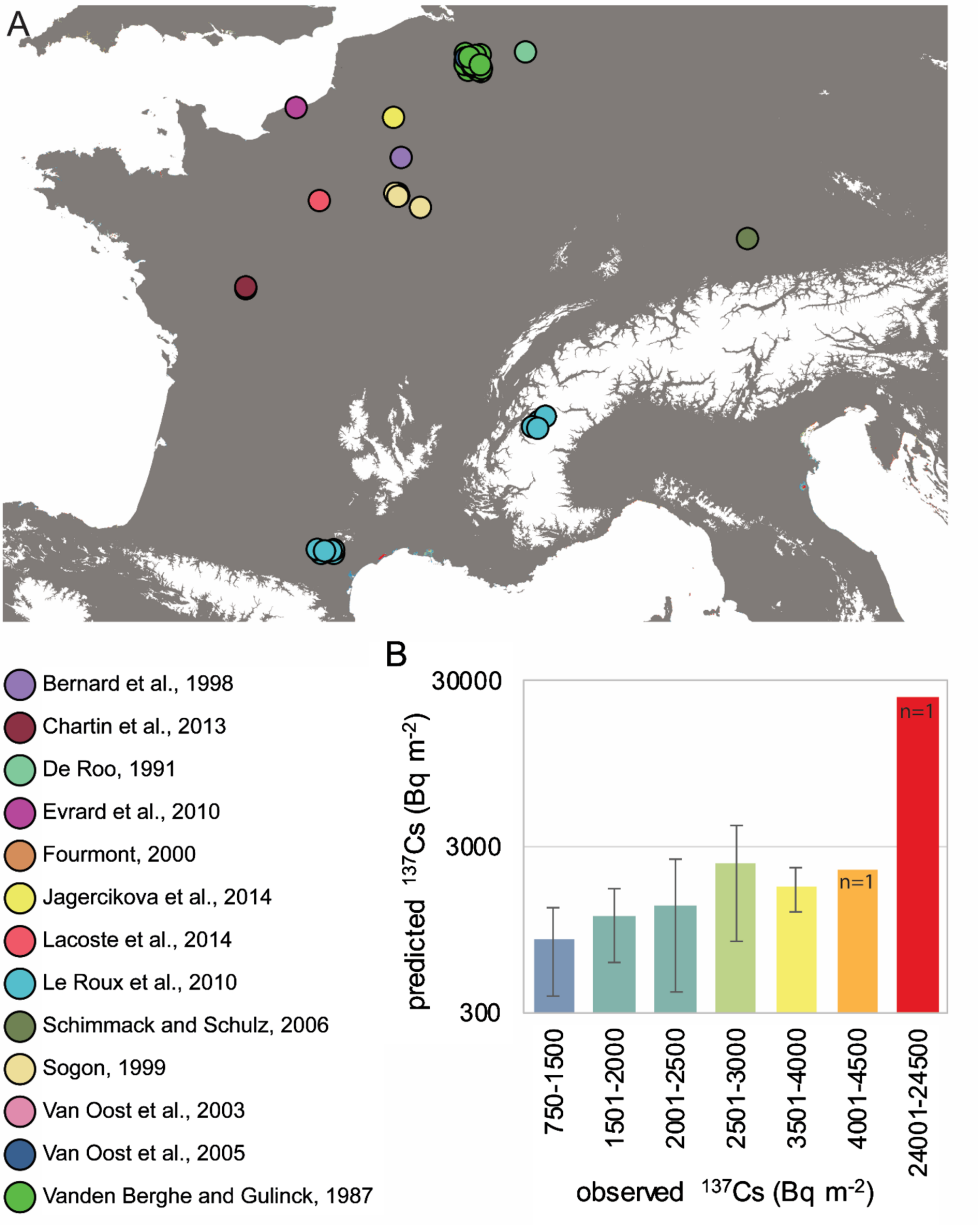
Locations of baseline ^137^Cs inventories reported in the literature^37–49^ (**A**) and comparison with those predicted in the current research (**B**). In **B**, the studies were grouped according to the colour scale applied in the ^137^Cs baseline map. All values decay-corrected to August 1, 2009. The map (projection: ETRS89-Lambert Azimuthal Equal Area) is own compilation, using ESRI ArcGIS 10.5 Desktop. The national boundaries are Intellectual Property from European National Mapping and Cadastral Authorities and is licensed on behalf of these by EuroGeographics. Original product is available for free at www.eurogeographics.org. Terms of the licence available https://eurogeographics.org/maps-for-europe/open-data/topographic-data/. Areas above 1,000 m a.s.l. have been masked (white) using the publicly available SRTM digital elevation model at 3 arcsec (https://www2.jpl.nasa.gov/srtm/).

For post-Chernobyl ^137^Cs inventories lower than 3,000 Bq m^−2^, assessments of local studies correspond well to the predicted inventory classes. However, for local baseline inventory assessments in the range of 3,000–4,500 Bq m^−2^, the model predictions are lower. Nonetheless, in the only local study conducted in a high Chernobyl fallout area, Schimmack and Schultz^37^ measured an inventory of 24,441 Bq m^−2^ that was predicted with 22,466 Bq m^−2^ in the current research. Overall, the predicted post-Chernobyl ^137^Cs inventories remained in a similar range as those published local baseline inventory assessments, despite the relatively coarse spatial resolution (500 m) of the predictions made in the current research (Fig. 8). The high small-scale variability of ^137^Cs inventories in soils that do not fulfil the reference site criteria is evident from the Radioactivity Environmental Monitoring (REM) database^50^. In Northern Italy, ^137^Cs measurements in 36 soil samples collected within a single grid cell of our map (predicted at 11,568 Bq m^−2^) range from 419 to 17,800 Bq m^−2^.

To conclude, the characterisation of ^240^Pu/^239^Pu atom ratios and ^239+240^Pu to ^137^Cs activity ratios in European soils provides an effective approach in order to quantify and map the spatial distribution of the Chernobyl derived ^239+240^Pu and ^137^Cs fallout in the environment and distinguishing it from other potential sources such as the global fallout. The applicability of this approach is supported by the progress made on the ICP-MS techniques to measure ^239+240^Pu (and both ^239^Pu and ^239^Pu individually) with a high sample throughput, compared to the long radiochemical sample preparation and counting that was required when conducting alpha spectrometry measurements. For the first time, it was possible to reveal the spatial global fallout pattern, which is more variable than previously expected. Although the magnitude of the global fallout was often neglected in the light of the Chernobyl fallout, when dated back to the respective major fallout years (1963 and 1986) the Chernobyl derived ^137^Cs fallout was 20% lower. The FRN baseline maps established for Western Europe based on this novel methodology provide a reference not only for quantifying FRNs levels in case of future additional accidental FRN fallout but also for providing a uniform reference map of radionuclide inventories for soil loss assessment. The FRN method is increasingly used to assess soil redistribution rates since the 1960s based on the comparison of ^137^Cs inventories measured at reference/undisturbed sites and those sites affected by soil redistribution including erosion and accumulation^51,52^. The assessment of the relative Chernobyl contribution is crucial to the application of ^137^Cs to quantify soil erosion rates^27^. However, given the short half-life of ^137^Cs, this well-established soil erosion tracer may be replaced by ^239+240^Pu as a surrogate in the future and, therefore, the ^239+240^Pu baseline map may gain further relevance with the expanding use of this tracer^53,54^. As such, the current research will provide a unique database for reconciling local investigations such as soil erosion studies conducted in individual hillslopes or catchments with European-wide homogeneous sample banks.

## Materials and methods

### Land use/cover area frame survey soil archive

Measurements of ^239+240^Pu and ^137^Cs have been conducted on soil samples collected in the framework of the Land Use/Cover Area frame Survey (LUCAS). LUCAS is an in-situ assessment, which means that the data was gathered through direct field observations across the EU Member States. In 2009, for the first time, a soil module was included in LUCAS. Topsoil samples (0–20 cm) were collected from 10% of the survey points, providing 19,857 soil samples across the 25 Member States. The topsoil samples were taken as composite samples, where a first subsample was taken at the location of the sampling point by sampling the topsoil (removing the litter) to a depth of 20 cm. Four other similar samples were then taken at a distance of 2 m from the original one following the four cardinal directions. The five subsamples were subsequently mixed together. This approach is effectively a physical averaging^55^ with the advantage of reducing the number of samples to analyse. In 2015, Switzerland joined the LUCAS program, and 160 samples were collected^56^.

The objective of the soil module in the LUCAS dataset was to improve the availability of harmonised data on soil parameters in Europe. All LUCAS soil samples were analysed in a single ISO-certified laboratory in order to avoid discrepancies arising from inter-laboratory differences^57^. Analysis of the soil parameters followed standard procedures (ISO 11,464 2006; ISO 10,694 1995; ISO 11,277 1998). The results, stored in the LUCAS topsoil database^58^ include (among others) the particle size distribution expressed as percentages of clay (0.0002—0.002 mm), silt (0.002- 0.05 mm), sand (0.05–0.2 mm) as well as organic carbon (%) and percentage coarse material (> 2 mm).

### Selection of baseline sites

The basis of the baseline inventory maps are ^239+240^Pu and ^137^Cs point measurements of the LUCAS soil samples. However, not all sites covered by LUCAS can be considered as undisturbed and as such, having preserved the initial FRN fallout signature. Via a geospatial database query potentially undisturbed reference sites have been selected. Potential reference sites fulfilled the criteria of having a slope angle < 2 degrees, being located outside of riparian zones and having remained under permanent grassland cover. Approximately 900 samples of the entire LUCAS dataset fulfilled these conditions. Subsequently, a stratified sampling using four rainfall classes along a rectangular grid was applied to select the final 165 samples. The mean annual rainfall map is the primary independent variable for the prediction of the baseline inventories. Through stratification by rainfall, we ensured that the full range of reference inventories are covered. Since the identification of reference sites is not always straightforward, the relationship between rainfall and inventory was also used to identify outliers (unreliable reference sites affected by disturbance) as reported by Chappell et al.^59^.

The most critical condition is to assess the permanence of grassland cover since the main FRN fallout period. This was checked by Landsat imagery since the 1970s and aerial photographs.

### Mass spectrometry for Pu atom ratio and concentration measurements

Prior to mass spectrometry analysis, the samples were ground, dry-ashed and spiked wit h ~ 0.005 Bq of a ^242^Pu yield tracer (obtained as a licensed solution from NIST). Pu was leached with 16 M nitric acid overnight at 80 °C and was subsequently separated from the leach solution using a Pu-selective TEVA resin^60^. The masses of ^239^Pu and ^240^Pu present in the sample were determined by isotope dilution calculations and then converted into the summed ^239+240^Pu activity.

The analysis was performed using a Thermo X7 quadrupole ICP-MS system located at Universidad de Cádiz. The instrument was used with a CETAC U-5000AT ultrasonic nebulizer system, and peristaltically pumped uptake rate of 0.30 mL/minute. Tuning the instrument with a solution of 0.2 μg/L uranium, a sensitivity of ~ 300,000 cps for ^238^U was achieved, along with background count rates of < 1 cps at m/z 239 and 240. The UH^+^/U^+^ was measured periodically (by monitoring signals at m/z 238 and 239) throughout the analytical runs using the U tuning solutions as well as U-spiked sample fractions for samples devoid of detectable Pu and was found to be 0.000080. The Q-ICP-MS exhibits a mass bias factor of 1.007 per m/z, favouring light masses, and this factor was used in correcting raw ratio data. For the analysis, a “peak-jump” routine was utilized with a 10 ms dwell time at m/z 238, 239, 240, and 242. An individual 46 s integration consisted of 1,000 sweeps through these four masses; two sequential integrations were acquired for each sample solution. A rinse solution consisting of 0.015 M ammonium oxalate dissolved in 0.1 M HNO_3_ was used, as needed, to rinse the sample introduction system, and signals at m/z 239 were monitored to ensure adequate decontamination of the sample introduction system prior to analysing the next sample. For 25 samples, the ^240^Pu/^239^Pu atomic ratio could not be measured with enough precision.

### Gamma spectrometry for ^137^Cs activity measurement

The activities of ^137^Cs were measured at two independent laboratories: the Laboratoire des Sciences du Climat et de l’Environnement (LSCE) in Gif-sur-Yvette, France and the Soil and Water Management & Crop Nutrition Laboratory of the Joint FAO/IAEA Division located in Seibersdorf, Austria. The activities of ^137^Cs were determined at the 662 keV emission peak by gamma spectrometry using very low-background coaxial ‘N’- and ‘P’-type high purity germanium detectors (Ortec, Canberra). Internal and certified International Atomic Energy Agency (IAEA) standards were used to verify counting efficiency and measurement reliability.

The measurements conducted at both laboratories were very well correlated (R^2^ > 0.99), and for all subsequent evaluation, the ^137^Cs activities corresponding to the estimated mean of both laboratories were used. All ^137^Cs activities expressed in Bq kg^−1^ were decay corrected to the 1st of August 2009, which corresponds to the average date of the soil sampling. In none of the samples, ^134^Cs was detected.

### Calculation of inventories and global fallout derived ^137^Cs inventory

Applying a decay correction to 1st of August 2009 the ^239+240^Pu/^137^Cs ratio of studies that reported exclusive global fallout (listed in Table 1) corresponds to 0.038 ± 0.004 SD. The inverse ^137^Cs/^239+240^Pu ratio equals 26.2 ± 2.3. The ^137^Cs activity in Bg kg^−1^ that originated from the global fallout (^137^Cs_Glob_ act) can be calculated as:

1 $$^{137} Cs_{Glob} act \, =^{239 + 240} Pu_{Glob} act \times 26.2$$

The ^239+240^ Pu activities and the decay-corrected ^137^Cs activities from different sources (*act*, Bq kg^−1^), were converted into inventories (*Inv,* Bq m^−2^) with the following equation:

2 $$Inv = act \times BD*\left( {1 - fcoarse/100} \right) \times d \times 10$$

where *BD* is the bulk density (kg m^−3^), which was derived from the LUCAS based bulk density map49, *fcoarse* represents the fraction of coarse material (> 2 mm, in per cent) and *d* the soil depth (m) of the sample. The Chernobyl derived inventory was established by subtracting the global fallout fraction from the total ^137^Cs inventory.

### Spatial model approach and support covariates

Generalized additive models (GAM) were fitted to the measured FRN inventories. GAMs are a generalization of linear regression models and can account for non-linear relationships by coefficients that can be expanded as smooth functions of covariates^62^. The smooth terms are modelled by splines. In this case, we used thin-plate splines with penalization. Geographic coordinates (x and y) are modelled as a 2d spline.

GAMs specify a distribution for the response variable *Y* and use a link function g relating the conditional mean μ (*Y*) of the response variable to an additive function of the predictors. Here, we used the Gaussian distribution with a log link. Thin plate regression splines were fitted by restricted maximum likelihood (REML) to prevent overfitting^63^. Geographic coordinates (x and y) are modelled as a 2d spline. We followed a backward stepwise approach to select the best set of covariates and to determine the relative influence of each of the covariates on the overall prediction capabilities of the model^64^. concurvity was checked, and covariates with high concurvity were excluded to prevent the presence of collinear covariates. The Akaike Information Criterion (AIC)^65^, and the deviances explained were calculated and compared for each of the models created.

The FRN inventories were then predicted at unsampled locations by applying the fitted model to the spatially continuous covariate layers. Predicted maps were prepared, with areas above 1,000 m altitude masked out because the LUCAS sampling did not include topsoil samples above 1,000 m a.s.l. and therefore to avoid unconstrained extrapolation, these areas were excluded. The standard error, which shows the theoretical range of deviation in the prediction made by the model, was calculated for every pixel of the created map.

As evidenced by several studies, FRN inventories are strongly correlated with precipitation and latitude (UNSCEAR). Climatic covariates of interest were obtained from the WorldClim (https://www.worldclim.org/) dataset. WorldClim data layers are the interpolated values of average monthly climate data collected from numerous weather stations worldwide during the period 1950–2000. The WorldClim spatial data layers have been established by a thin plate smoothing spline with latitude, longitude and elevation as independent variables to locally interpolate the station data^66^. From the WorldClim dataset, we also used minimum and maximum temperature and the monthly averages as covariates. Two additional rainfall datasets were used to target the main fallout periods. To capture the Chernobyl fallout more precisely, we used daily rainfall maps of the period from April 28th to May 6^th^ in 1986 (Supplementary Fig. 4). These maps with a rather coarse resolution were derived from the Gridded Agro-Meteorological Data in Europe that contains meteorological parameters from weather stations interpolated on a 25 × 25 km grid^67^ Meteorological data are available on a daily basis from 1975 to the last calendar year completed, covering the EU Member States, neighbouring European countries, and the Mediterranean countries. Further, we used the dataset Climatologies at high resolution for the Earth’s land surface areas (CHELSA) to reconstruct the precipitation during the main global fallout periods from 1952–1980 and the more intense period from 1959–1963^68^. The CHELSA data (3 arcsec spatial resolution) results from a downscaled ERA-interim model and includes topoclimate such as orographic rainfall and wind fields (Supplementary Fig. 5).

The NASA-SRTM digital elevation model (3 arc-second resolution equivalent to 100 m at European latitudes) was used to derive valley height. Another main set of covariates originated from Moderate Resolution Imaging Spectroradiometer (MODIS). We use the first principal component analysis of the MODIS red band and the near-infrared band for the year 2009 as a proxy for the vegetation status (Supplementary Fig. 6). The environmental covariates used to predict the different FRN inventories in this study are listed in Supplementary Table 1.

A spatial resolution of 500 m was selected as a compromise between the different original resolutions of the covariates and to take the uncertainty of the covariates themselves into account. All statistical analysis has been carried out in Rx64 3.6.1 using the packages sf, raster, gstat and mgcv. For the layout of the maps, we used ArcGIS (ESRI) version 10.7 and Adobe Illustrator CC 2017.

### Uncertainty of the plutonium based approach

The validity of the proposed approach to reconstruct the baseline map of global and Chernobyl fallout relies on the careful selection of the undisturbed soil sites. However, despite all our efforts, some of the inventories could have been affected by local soil disturbance. For those sites affected by such small-scale disturbances, we expect a low prediction accuracy and high residuals associated with the GAM model outputs. Although the high residues show no remaining spatial autocorrelation for ^137^Cs and ^239+240^Pu and are spread non-systematically across the study area (Supplementary Fig. 6), the inclusion of some disturbed sites in the dataset cannot be entirely excluded. The relative standard error (RSE) for the ^239+240^Pu baseline and the global fallout map is lower than 20% for the major part of the study area (Supplementary Fig. 8). Zones of high uncertainty are the Alps and the Mediterranean coast. For the total and Chernobyl derived ^137^Cs, RSE is generally higher, particularly in the western part of France.

Another potential source of uncertainty associated with the approach is related to the sampling depth. For the LUCAS survey, samples have been taken down to a depth of 20 cm, and the lower part of the soil profile was not collected. Accordingly, the fraction of the total FRN inventory contained in deeper layers was not considered in this study. However, as observed in multiple studies, ^239+240^Pu, as well as ^137^Cs, are strongly adsorbed by the mineral soil and their concentrations, therefore typically decline exponentially with soil depth. For ^239+240^Pu, a review of published ^239+240^Pu depth profiles shows that 95% of the Pu inventory is stored in the upper 20 cm of the soil^53^. Similarly, for ^137^Cs, multiple studies describe the exponential decline of the activities and related inventories with soil depth^51,69^. Studies using both FRNs generally calculated a higher downward migration of ^239+240^Pu compared to ^137^Cs^26^. However, it was demonstrated that the more recent Chernobyl ^137^Cs fallout remains concentrated in the uppermost cm of the soil profile^27^.

FRN inventories are commonly assessed by multiplying the activity by the bulk density of the fine (< 2 mm) soil material. However, bulk density was not available for the LUCAS composite samples. Therefore, inventory calculations were based on the interpolated map of bulk densities and the fraction of coarse material (measured in classes) to convert FRN mass activities into inventories^61^. However, it is assumed that overall the sources of uncertainties leading to an underestimation of the inventories (e.g. sampling of the 0–20 cm soil layer rather than the entire profile) will balance those leading to an overestimation of these values. The activity ratios and the separation of global versus Chernobyl fallout are affected to a minor degree by the sampling protocol. The activity ratios may be impacted by differences in the migration velocity and the migration time of the different fallout sources, which might lead to some variability of the ratio with soil depth as was observed for an alpine soil with Chernobyl derived ^137^Cs^27^. However, the mixing of the composite soil samples and the consideration of a single 0–20 cm homogenized sample is expected to smooth out the small scale deviations that might be observed from the detailed analysis of the full set of thinner soil layers across the profiles.

## Supplementary information

Supplementary file1 (DOCX 6219 kb)

## Data Availability

The authors declare that all other data supporting the findings of this study are available in the European Soil Data Centre (ESDAC): https://esdac.jrc.ec.europa.eu/themes/plutonium-and-cesium-inventories-european-topsoils.
