# Supplementary material for: Plutonium aided reconstruction of caesium atmospheric fallout in European topsoils
Source: Sci Rep. 2020 Jul 16;10:11858. doi: 10.1038/s41598-020-68736-2 (PMC7366645; doi:10.1038/s41598-020-68736-2)
Supplement: Supplementary file 1 — Supplementary file1 (DOCX 6219 kb) [file 41598_2020_68736_MOESM1_ESM.docx]

Plutonium aided reconstruction of caesium atmospheric fallout in European topsoils

Katrin Meusburger*^1^, Olivier Evrard^2^, Christine Alewell^3^, Pasquale Borrelli^3^, Giorgia Cinelli^4^, Michael Ketterer^5^, Lionel Mabit^6^, Panos Panagos^4^, Kristof van Oost^7.8^, Cristiano Ballabio^4^

Contributors:

1. Swiss Federal Institute for Forest, Snow and Landscape Research (WSL), CH-8903 Birmensdorf, Switzerland.
2. Laboratoire des Sciences du Climat et de l’Environnement (LSCE-IPSL), UMR 8212 (CEA-CNRS-UVSQ), Université Paris-Saclay, CEA Saclay, l’Orme des Merisiers, 91191 Gif-sur-Yvette Cedex, France.
3. Environmental Geosciences, University of Basel, Bernoullistrasse 30, CH-4056 Basel, Switzerland.
4. European Commission, Joint Research Centre, Via E. Fermi 2749, I-21027 Ispra (VA), Italy.
5. Chemistry Department, Metropolitan State University of Denver, Colorado, USA.
6. Soil and Water Management & Crop Nutrition Laboratory (SWMCNL), Joint FAO/IAEA Division of Nuclear Techniques in Food and Agriculture, Seibersdorf, Austria.
7. TECLIM, George Lemaitre Center for Earth and Climate, Earth and Life Institute, Université Catholique de Louvain, Louvain-La-Neuve, Belgium.
8. Fonds de la Recherche Scientifique (FNRS), Brussels, 1000, Belgium.

# Supplementary information

Supplementary Table 1 Covariates used for each spatial model.

| Target variable | Covariates | R-sq.(adj.) |
| --- | --- | --- |
| total Cs-137 inventory | XY/ prec_52_80/ prec_05_1986/ nir_PCAb4/ red_PCAb1/ val_height | 0.96 |
| Chernobyl Cs-137 inventory | XY/ prec_05_1986/ prec_M2/ prec_M3/ nir_PCAb4/ val_height | 0.97 |
| Global Cs-137 inventory | XY/ prec_52_80/ prec_12/ nir_PCAb4/ tmin_11/ val_height | 0.70 |
| total Pu-239+240 inventory | XY/ prec_52_80/ prec_12/ nir_PCAb4/ tmin_11/ val_height | 0.70 |


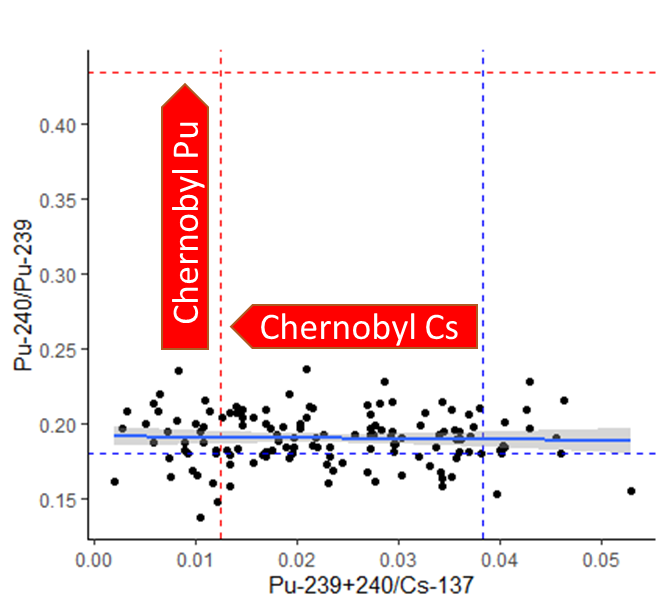


Supplementary Figure 1 Pu-240 to Pu-239 atom ratio against Pu-239+240/Cs-137 activity ratio. The intersection of the red dashed lines would indicate exclusive Chernobyl-derived fallout for Pu-239+240 and Cs-137. The intersection of the blue dashed lines would indicate exclusive global-fallout derived fallout for Pu-239+240 and Cs-137. The actual sample distribution (black dots) shows no significant trend (blue line) of increasing Chernobyl-derived Pu-239+240 with increasing Chernobyl-derived Cs-137 contribution.


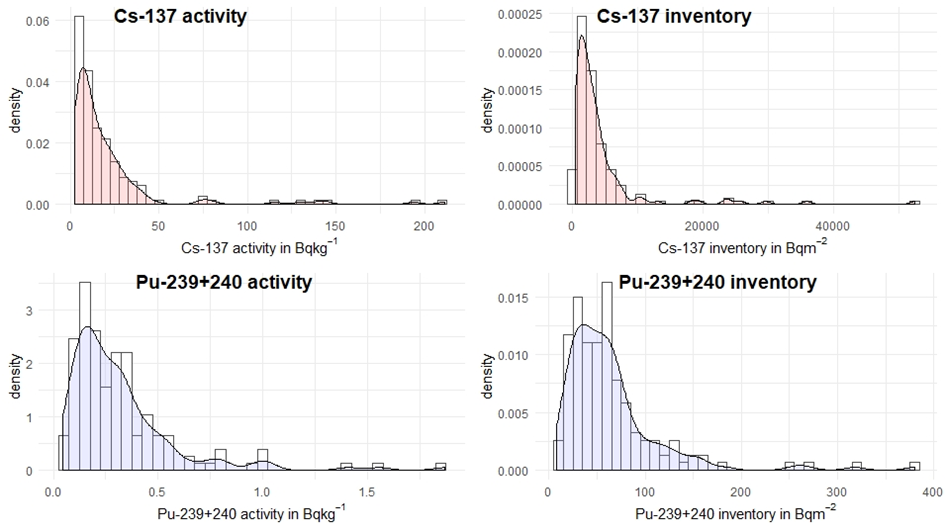


Supplementary Figure 2 Kernel density plot of measured ^239+240^Pu and ^137^Cs mass activities and derived inventories of European topsoil samples (0-20 cm; n = 160).


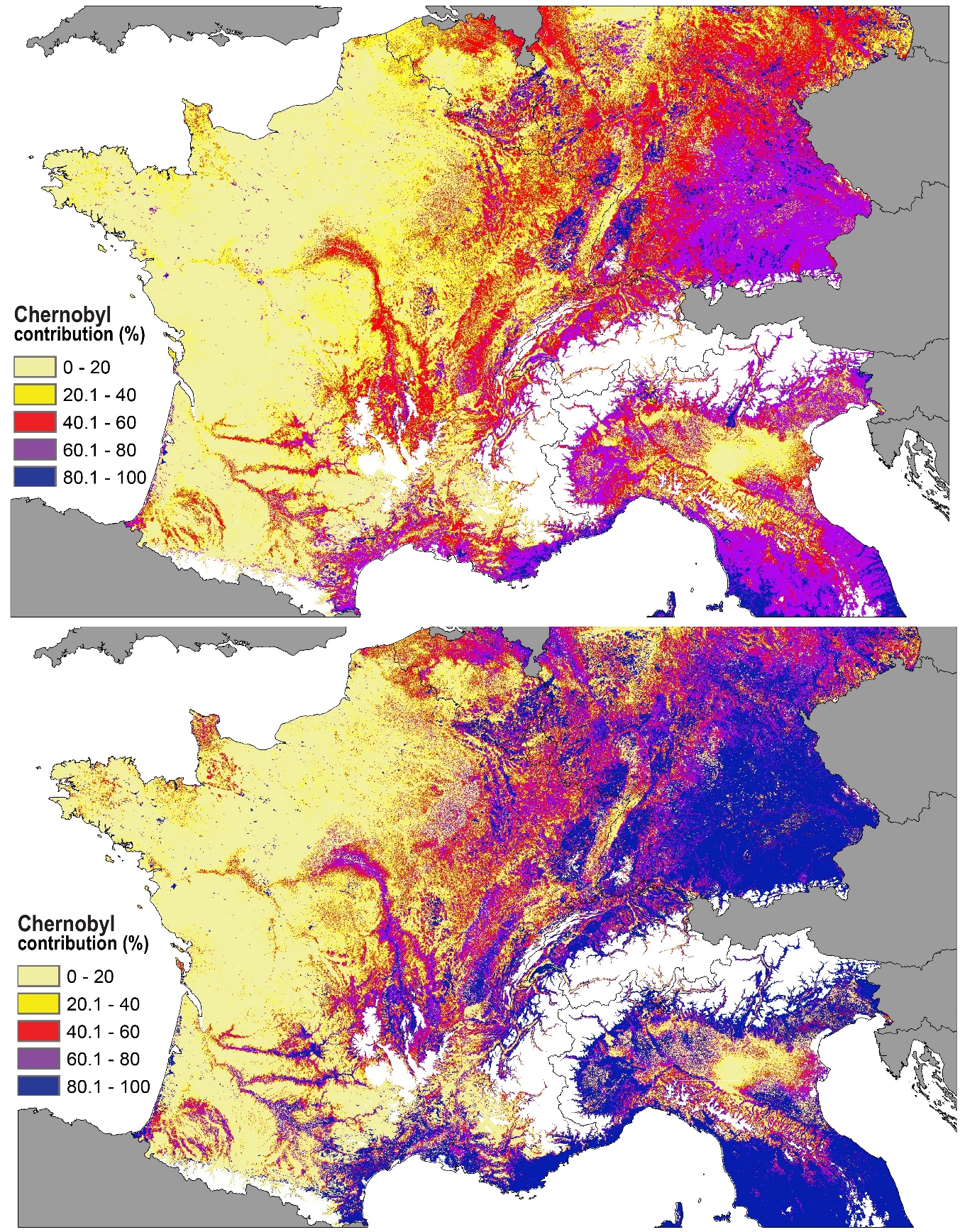


Supplementary Figure 3 Minimum (upper panel) and maximum (lower panel) scenario of Chernobyl-derived ^137^Cs fallout contribution (%) of topsoil (0-20cm) samples decay-corrected to August 1, 2009. Areas above 1,000 m a.s.l. have been masked (white) using the publicly available SRTM digital elevation model at 3 arcsec (https://www2.jpl.nasa.gov/srtm/). The map (projection: ETRS89-Lambert Azimuthal Equal Area) is own compilation, using ESRI ArcGIS 10.5 Desktop. Authorities and is licensed on behalf of these by EuroGeographics. Original product is available for free at [www.eurogeographics.org](http://eurogeographics.org/). Terms of the licence available <https://eurogeographics.org/maps-for-europe/open-data/topographic-data/>.


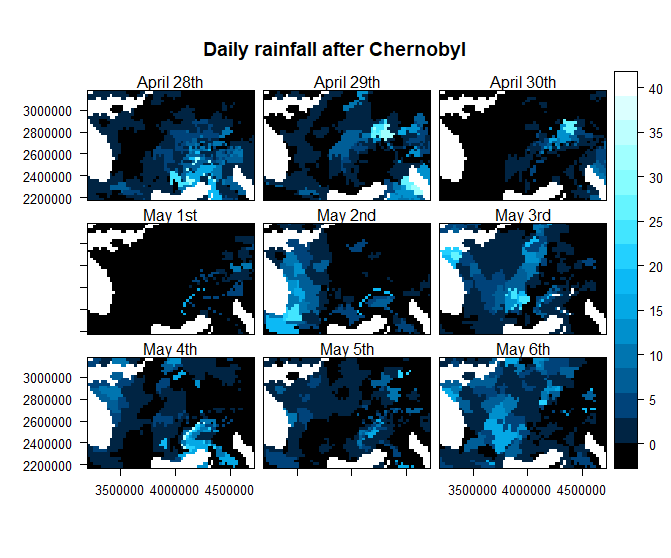
Supplementary Figure 4 Daily rainfall (mm) after the Chernobyl accident. April 28th to May 6th corresponds to the 28.4.1986 to 6.5.1986. The maps were derived from the CGMS database, which contains meteorological parameters from weather stations interpolated on a 25x25 km grid^52^. The grid labels refer to the coordinates in ETRS89-Lambert Azimuthal Equal Area (m).


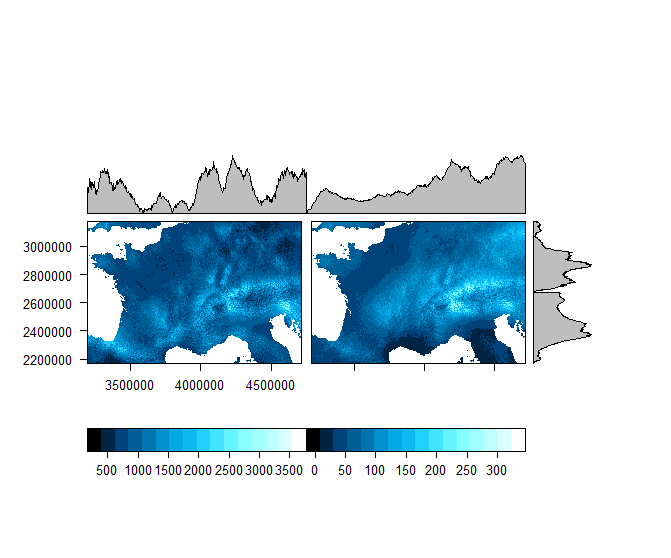


Supplementary Figure 5 Average annual rainfall (mm) during the main global fallout from 1952 to 1980 (left) and average monthly rainfall (mm) in May 1986. Both maps are derived from the dataset Climatologies at high resolution for the earth’s land surface areas (CHELSA)^53^. The grid labels refer to the coordinates in ETRS89-Lambert Azimuthal Equal Area (m).


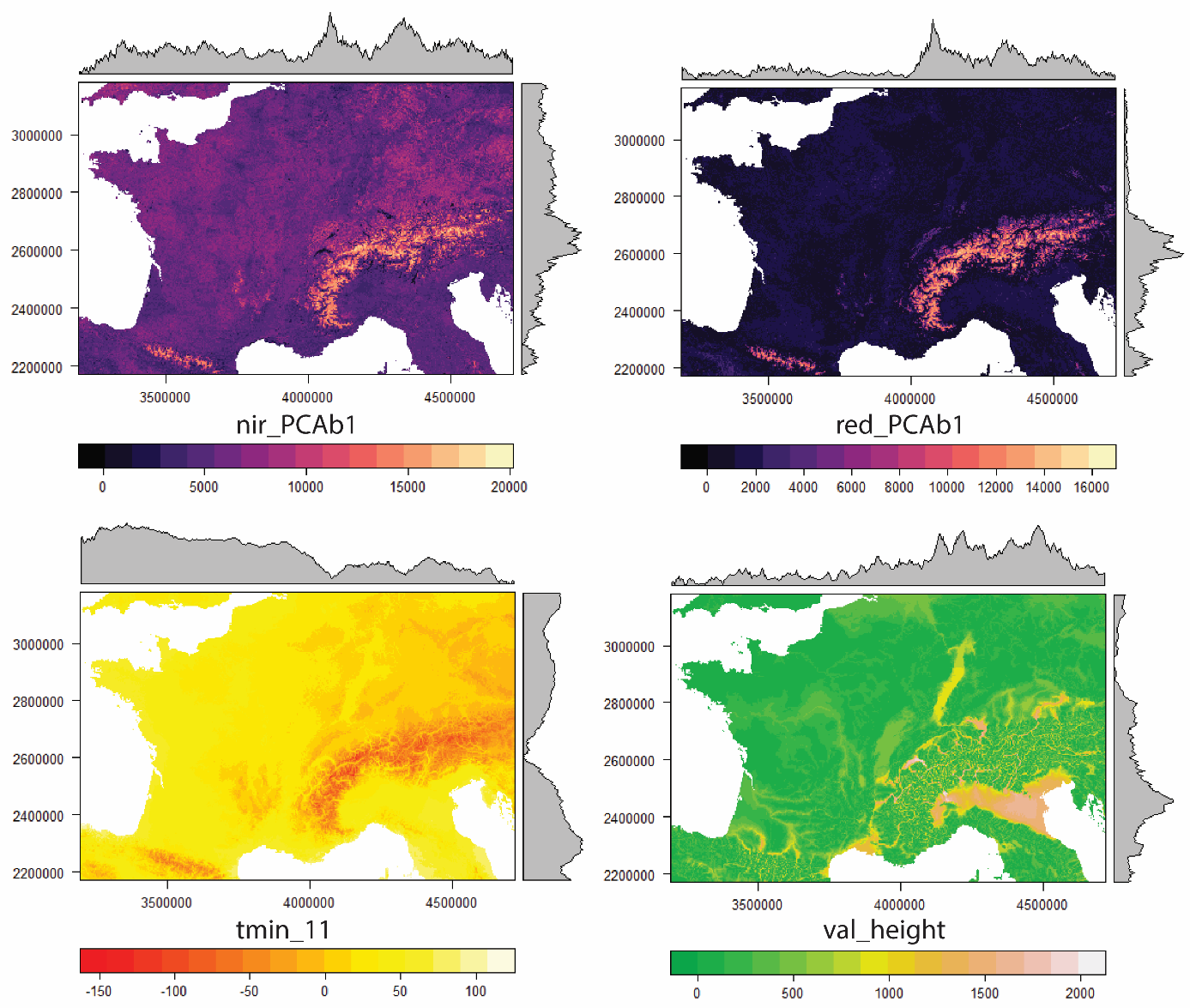


Supplementary Figure 6 Spatial covariates used to predict ^137^Cs and ^239+240^Pu fallout patterns: the first principal component analysis of the MODIS near-infrared (nir_PCAb1) and red (red_PCAb1) band for the year 2009 as a proxy for vegetation status, the average minimum temperature in November (tmin_11) derived from WorldClim and the valley height (val_height) derived from NASA-SRTM digital elevation model at3 arc-second resolution equivalent to 100 m at European latitudes (https://www2.jpl.nasa.gov/srtm/). The grid labels refer to the coordinates in ETRS89-Lambert Azimuthal Equal Area projection (m). Histograms of the maps are displayed along the right and top side of each map.


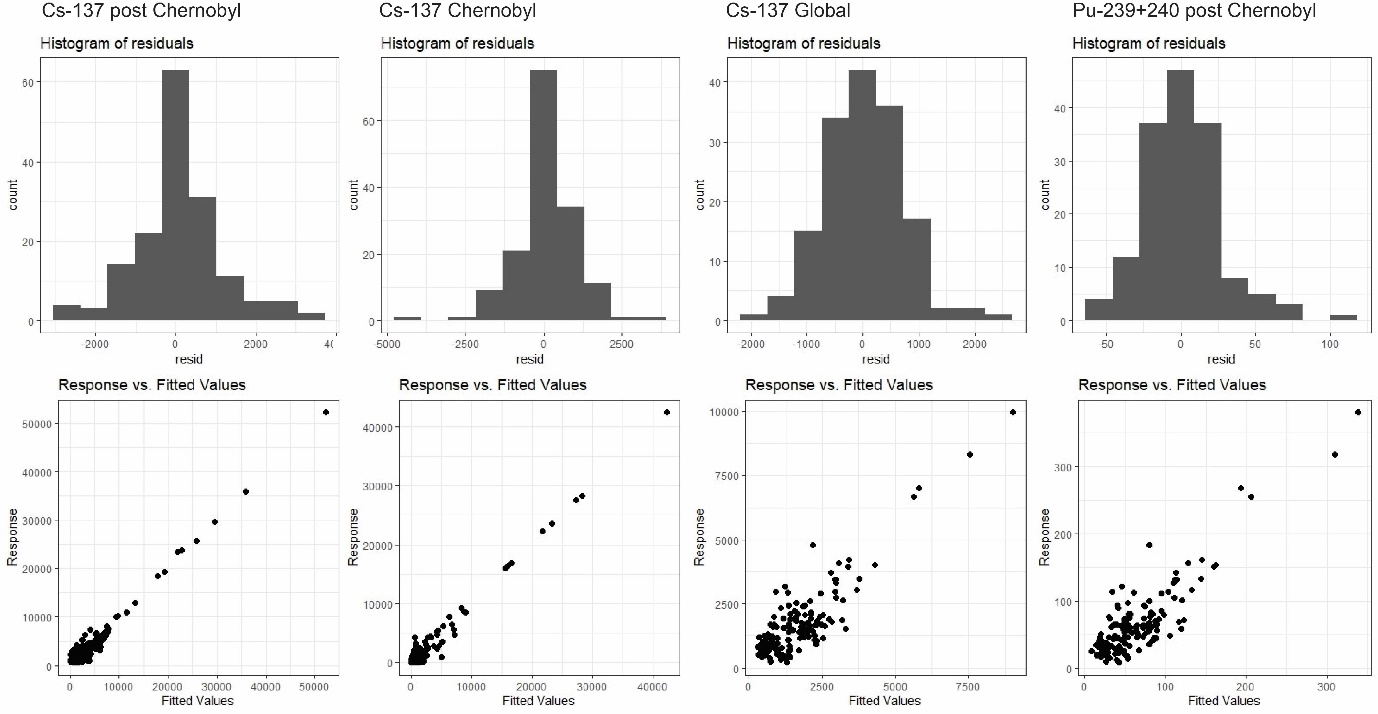


Supplementary Figure 7 Histograms of residuals (above) resulting from measured (response) versus fitted inventories of ^137^Cs and ^239+240^Pu separated for the global and Chernobyl sources.


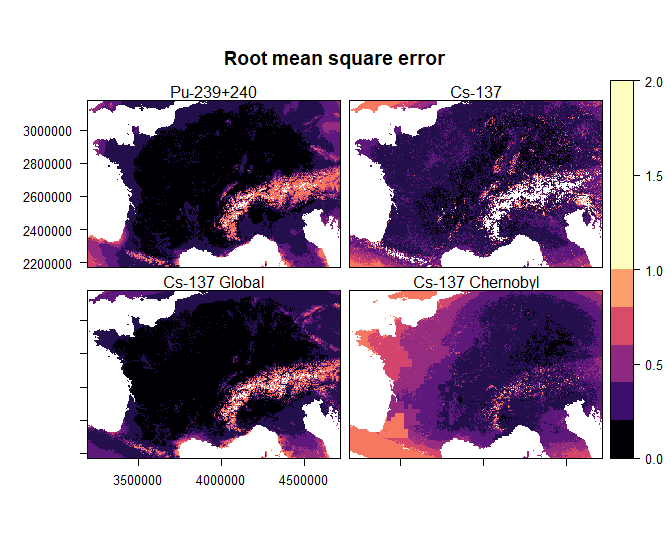


Supplementary Figure 8 Root mean square error for the ^239+240^Pu and the ^137^Cs baseline (top) and the global- and Chernobyl-derived ^137^Cs. The grid labels refer to the coordinates in ETRS89-Lambert Azimuthal Equal Area projection (m).
